# Study on Improvement of Welding Technology and Toughening Mechanism of Zr on Weld Metal of Q960 Steel

**DOI:** 10.3390/ma13040892

**Published:** 2020-02-17

**Authors:** Xingyu Ai, Zhengjun Liu, Dan Wu

**Affiliations:** Department of Material Science and Engineering, Shenyang University of Technology, Shenyang 110870, China

**Keywords:** Q960 high strength steel, self-shielded flux-cored wire, alloy agent, acicular ferrite, strengthening and toughening mechanism

## Abstract

Q960 high-strength steel is widely used in pressure vessels, bridges, offshore platforms and other important steel structural components because of its high strength and good plastic toughness, but alloy elements added to this kind of steel have strong hardenability, especially after welding, so the strength and toughness cannot meet the requirements, which limits its application in a wider range. In this paper, from the point of view of the metallurgical treatment of the weld, the goal is to improve the strength and toughness of the Q960 high strength steel weld metal In order to analyze the influence of Zr on the welding process of Q960 steel and the strengthening and toughening effect of weld metal, this paper takes Fe-Mn-Mo-Cr-Ni as the main alloy system, BaF_2_-CaF_2_-Al-Mg as the basic slag system, and adopts the method of melting consumable electrode self-shielded for welding, and analyzes the welding process, microstructure, tensile property and impact toughness of the welded joint. The experimental results show that when the weld metal contains 0.0061% Zr, the minimum spatter rate is only 7%, the maximum slag removal rate is 95%, the maximum hardness is 357HV, the maximum elongation is 34%, and the impact toughness is the highest. At this time, the acicular ferrite content in the weld microstructure is the highest, and there is a certain amount of equiaxed fine-grained ferrite, and the content of proeutectoid ferrite is the least, which effectively improves the strength and toughness of the weld metal.

## 1. Introduction

High strength low alloy (HSLA) steel is widely used in pressure vessels, bridges, offshore platforms and other large-scale structures because of its high strength and good plasticity, toughness [1,2,3,4,5]. With the continuous emergence of new materials and processes, and the continuous improvement of manufacturing technology and molding technology, "energy saving, environmental protection and material reduction" has become the development goal of the machinery industry, especially the demand for light weight is becoming more and more urgent [6,7,8,9,10,11]. In construction machinery products, 50% ~ 70% of the steel needs to be welded. The quality of the welding structure directly determines the product quality and the safety and reliability of the components [11,12,13,14]. However, a large number of alloy elements are added into the smelting process of low alloy high-strength steel, which leads to a greater hardenability. After welding, weldability problems such as cracks, embrittlement and softening of heat affected zone easily appear, and especially the low-temperature impact toughness is not up to the requirements, which limits the wide use of the low-alloy high-strength steel in a wider range of applications [13,14,15,16].

The results show that the higher melting point of oxide, the more obvious obstruction to austenite grain growth, and can promote acicular ferrite nucleation and improve the impact toughness of weld metal [17]. Zr is a strong carbon, nitrogen and oxide-forming element. The oxide forming energy of Zr is lower than that of Ti, and the melting point of Zr is higher than that of Ti. As a microalloy element, Zr has been studied a lot in the influence of microstructure and properties of steel [18], but studies on the influence of Zr on the microstructure and properties of weld metal are relatively few.

Self-shielded flux cored wire is a new type of welding material which can be welded without additional protective measures. It has the characteristics of strong wind resistance, simple welding equipment and suitable for field construction, so it can weld high-quality welds. In this paper, taking Fe-Mn-Mo-Cr-Ni as the main alloy system and BaF_2_-CaF_2_-Al-Mg as the basic slag system, the influence rule and mechanism of Zr element in welding wire powder on the microstructure and mechanical properties of weld metal were studied, and the strengthening and toughening mechanism of weld metal of high strength steel was explored.

## 2. Materials and Methods

The steel plate for the test is Q960, a low-carbon quenched and tempered steel produced by Wuyang Iron and Steel Co., Ltd. (Wugang, China), with a thickness of 10 mm. Its main chemical composition is shown in Table 1, and its mechanical properties are shown in Table 2. The steel strip used for the test is Steel Plate Cold Commercial (SPCC) steel of the H08A brand, which is produced by Shanghai Baosteel (Shanghai, China) and made by a cold rolling process. The width of the steel strip is 10 mm and the thickness is 0.3 mm. Its chemical composition is shown in Table 3. 

The metal powder for making flux cored wire contains electrolytic manganese powder, nickel powder, molybdenum powder, metal chromium, aluminum powder, zirconium powder and iron powder, and the particle size is between 80–100 mesh. Mn, Si, Ni, Mo, Cr, Ti, B, Al are often used as self-shielded flux cored wires for welding of high strength steel. Mn can react with S, the product of MNS, is helpful to prevent hot cracks caused by FES inclusions. Cr, Si, B can form strong carbide with C, which increases the hardenability and strength of weld. Ni and Mo can shift the transition curve of continuous cooling of weld metal to the right and promote the formation of acicular ferrite. Ti, B can promote the nucleation and growth of acicular ferrite and improve the impact toughness of weld metal. The addition of Al can greatly reduce the O and N content and porosity sensitivity [3,4,5]. In this experiment, the content of other powder is not changed, only the content of Zr powder is adjusted. The formula of the powder is shown in Table 4.

Through chemical analysis, the content of Zr in weld metal is 0%, 0.0028%, 0.0061%, 0.0087% and 0.0135% respectively, and the composition of other weld metal alloy elements is shown in Table 5. The slag system of flux cored wire is BaF_2_-CaF_2_-Al-Mg. The above metal powder and mineral powder are provided by Sichuan Kehui Industry Co., Ltd. (Chengdu, China). The composition of the metal powder is shown in Table 5.

**Figure 1:**
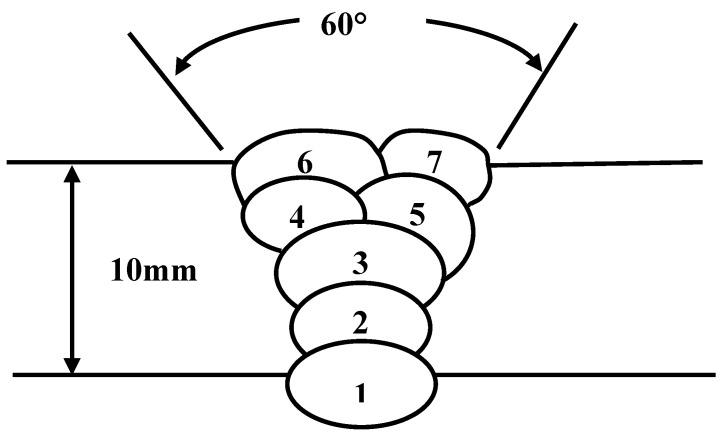
Diagram of a welded joint.

The production of flux cored wire powder core needs to go through four processes: powder blending, powder baking, powder screening and powder mixing. The processed steel strip and powder core are sent to the wire forming machine, and the flux cored wire required for the experiment is obtained by drawing and reducing the diameter, with the diameter of 1.6mm. The powder filling rate is 35%. Welding is carried out with a self-shielded melting electrode. A YD-500 welder (Panasonic, Tangshan, China) is selected as the welding equipment. The welding position is flat welding. The number of weld layers is five layers and seven weld passes. The welding process parameters and the welding joint schematic diagram are shown in Table 6 and Figure 1, respectively.

**Figure 2:**
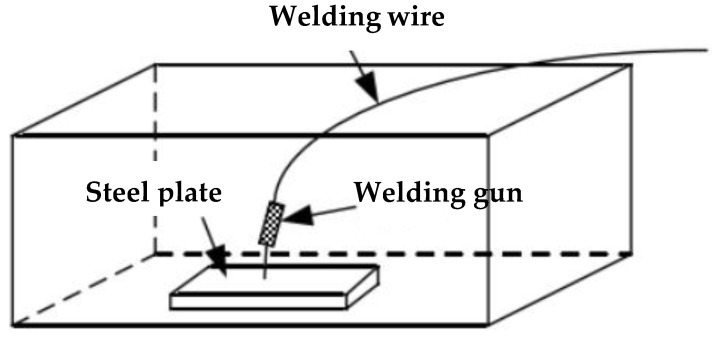
Schematic diagram of the welding spatter test equipment.

The spatter rate and slag removal should be considered in the welding process of self-shielded flux cored wire. The bad spatter rate will cause workpiece pollution, the low transition coefficient of alloy elements will affect the welding quality, the welding arc is unstable, and the welding deposit efficiency will be reduced. The low slag removal rate will increase the welder’s working strength, reduce the welding production efficiency, and make the weld metal prone to slag inclusion and other defects. The spatter rate was measured by quality analysis. A self-made welding spatter collection device is shown in Figure 2. A proper sprayed amount of anti-spatter agent on the inner wall of the device before welding will facilitate the collection of welding spatter after welding. A steel plate with 300 mm × 200 mm × 10 mm dimensions is placed on the platform inside the device, anti-spatter agent is applied on both sides of the steel plate groove, and the steel plate mass *m*_0_ is weighed at this time. The surface of the steel plate is welded with the parameters showed in Table 7, and the welding time is 1 minute. After welding, the spatter particles on the inner wall and the steel plate surface shall be collected and weighed *m*_1_ immediately. At the same time, the welded steel plate weight *m*_2_ is recorded. According to Equation (1), the spatter rate of welding is obtained:

(1) $$\mathrm{The}\;\mathrm{spatter}\;\mathrm{rate}\;=\frac{m_{1}}{m_{2}-m_{0}+m_{1}}$$

**Figure 3:**
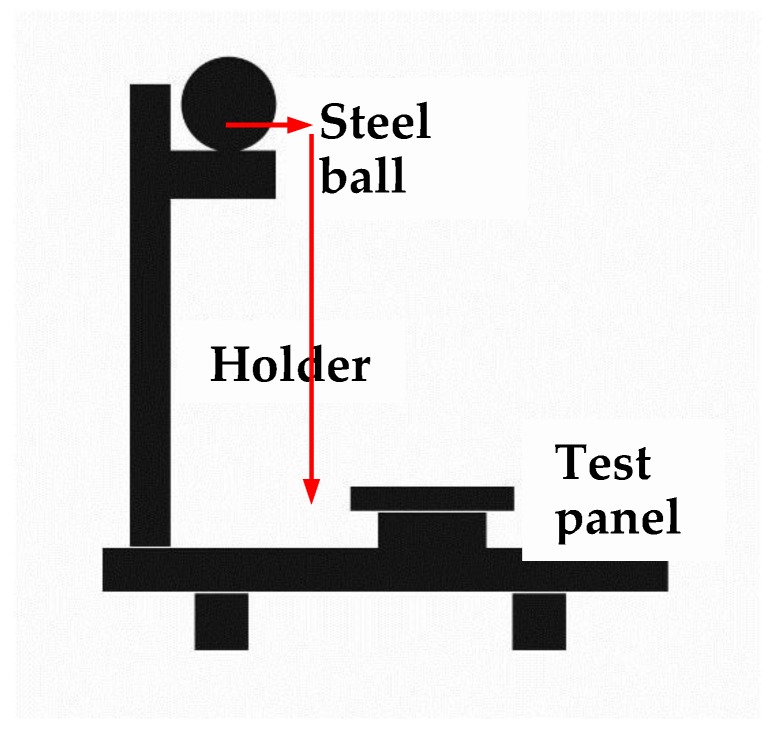
Schematic diagram of the ball drop test machine.

The drop hammer method is used to measure the slag removal of the welding seam. Deslagging treatment was carried out for the welded specimens by a falling ball test, and the test principle is shown in Figure 3. The weight of the steel ball is 3000 g, and the test time is 1 min after welding. The hammering position of the steel ball is the back of the steel plate corresponding to the weld, and the falling height of the steel ball is 500 mm.

The length of non-deslagging, serious slag sticking and slight slag sticking on the weld surface shall be counted and calculated according to Equation (2):

(2) $$D=\frac{l-\left(l_{0}+0.5l_{1}+0.2l_{2}\right)}{l}$$

where *D* is the slag removal rate of the weld; *l* is the length of the weld, mm; *l*_0_ is the length of the non-slag removal, mm; *l*_1_ is the length of the serious slag sticking, mm; *l*_2_ is the length of the slight slag sticking, mm.

The welded specimens are processed by wire cut electrical discharge machining (WEDM) to obtain metallographic specimens, transmission specimens, tensile test specimens and impact test specimens. 

The metallographic samples for OM and SEM observations were prepared by grinding using SiC abrasive papers from no.240 up to no.2000, mechanically polishing using 2.5 mm diamond abrasive and then chemical etching with a solution of 4% nital, and the etching time is 10 s ~ 15 s. The model of optical microscope used is a GX–51 (Olympus, Tokyo, Japan). The metallographic specimens, tensile fracture and impact fracture were analyzed by a S-3400N scanning electron microscope (Hitachi, Tokyo, Japan) and the phase composition of inclusions was analyzed by energy dispersive spectrometry (EDS). 

TEM discs with thickness at least 500 μm were cut from the gauge sections of the fractured specimens perpendicular to the loading axis. Discs of 3 mm in diameter were punched out after mechanically grinding to about 50 μm in thickness. Thin foils for TEM observations were prepared by twin-jet electropolishing in a solution of 10 vol % perchloric acid and 90 vol % ethanol at 20 V and −19 °C. The microstructure and microanalysis were carried out by TECNAI G2 F20 field emission transmission electron microscope.

The chemical composition of Zr, Mn, Si, Mo, Cr, Ni, Ti, B, Al in weld metal was analyzed by an ARL4460 photoelectric direct reading spectrometer (Thermo Fisher, Waltham, MA, USA). The content of O and N in weld metal was measured by ON 900 oxygen nitrogen analyzer (Eltra, Hamburg, Germany).

The hardness of weld metal was measured by a THVS-5 microhardness tester (Beijing Times peak Technology Co., Ltd, Beijing, China). The load was 500 g and the holding time was 15 s. The distance between the measurement position and the upper surface of the weld is 2 mm. Each specimen measured five points and the average value was obtained. Interval between points: 500 um, as shown in Figure 9.

The tensile fracture specimens and impact fracture specimens are only cleaned using acetone solution without any other treatment. Tensile tests were carried out according to the “low alloy steel flux cored wire” standard (GB/T 17493–2008). The test machine was a GNT 200 electronic universal testing machine (CNS, Shanghai, China). The tensile strength and elongation at fracture were measured, three parallel samples for each condition, and the average values of tensile strength and elongation were obtained. 

**Figure 4:**
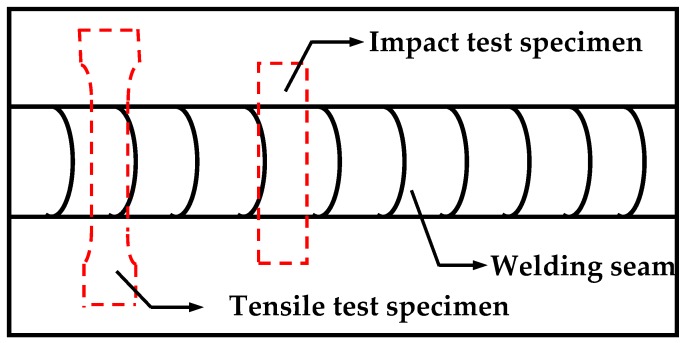
Schematic diagram of machining orientation of tensile and impact test specimens.

The weld zone and HAZ were machined into impact test specimens According to GB / T 17493-2008, the impact performance test shall be carried out at 25 °C, 0 °C; and −60 °C. The impact test was carried out on a JB-30B impact test machine (Kocheng testing machine Co., Ltd, Chengde, China). The size of test specimens is 10 mm × 10 mm × 55 mm, V-notches were prepared by an impact specimen notching broaching machine, model CSL-A (Liangong testing equipment Co., Ltd, Jinan, China). The notch position is the center of the test specimen, i.e., the center of the weld and the depth is 2 mm. The instantaneous pendulum speed is 5 m/s, 5 parallel samples for each condition, and the average values of impact energy were obtained. The impact test is carried out according to GB/T 2650-2008 “welding joint impact test method” standard. The machining orientation of tensile test specimens and impact test specimens is perpendicular to the weld, as shown in Figure 4. The dimensions of tensile and impact specimens are shown in Figures 10 and 12.

## 3. Results and Discussion

### 3.1. Effect of Zr Content on Spatter Rate and Slag Removal

**Figure 5:**
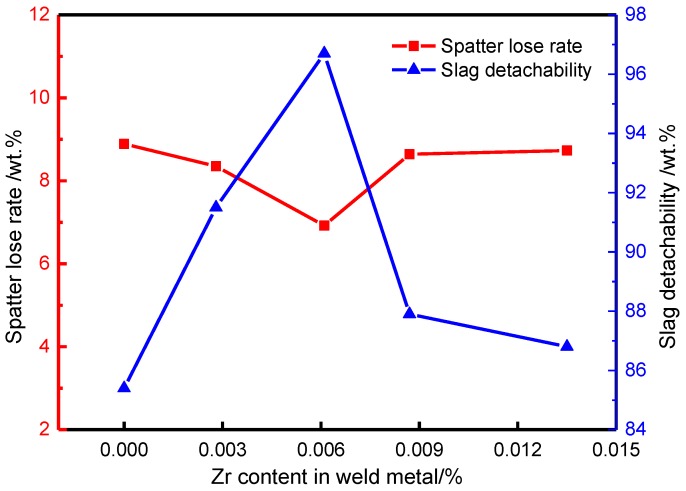
Effect of Zr contents on welding spatter rate and slag removal rate.

By means of chemical analysis, the content of Zr in weld metal is 0%, 0.0028%, 0.0061%, 0.0087% and 0.0135% respectively, and the composition of other weld metal alloy elements is shown in Table 7. Figure 5 shows the effect of Zr content of weld metal on the spatter rate and slag removal. It can be seen that with the increase of Zr element content, the welding spatter rate decreases gradually. When the weld metal contains 0.0061% Zr, the minimum spatter rate of weld metal is only 7%; with the increase of Zr content of weld metal, the welding spatter rate begins to increase again. The relationship between the slag removal rate and the Zr content in the weld metal is just the opposite to the spatter rate. When the Zr content in the weld metal reaches 0.0061%, the highest slag removal rate is 95%.

Zr is a strong reducing agent in the weld metal of high strength steel. When the Zr in flux cored wire is less (0.0028% Zr), small particle splashing easily occurs during the welding process because of the high O content and low surface tension of droplets. With the increase of Zr content in the flux cored wire, Zr will react with O to form ZrO_2_, which will reduce the O content in the droplet and ZrO_2_ has an ion bond. The bond energy is larger, which will increase the surface tension of the droplet. Therefore, with the increase of Zr content (0.0061% Zr), the surface tension of the droplet will be larger and larger, and the spatter rate of welding will be smaller. However, when the Zr content is too much (0.0135% Zr), the droplet will become coarse due to the excessive surface tension of the droplet, forming a large particle spatter, which will increase the spatter rate. Therefore, in this test, from the point of view of reducing the spatter rate, the Zr content of weld metal is set at 0.0061%.

The slag removal rate of the weld is related to the physical properties of the slag itself. In this experiment, ZrO_2_, the oxidation product of Zr, has different crystal structures at different temperatures [17,18,19]. At a temperature ~1170 °C, ZrO_2_ oxide shows monoclinic structure; at 1170 °C ~ 2370 °C, ZrO_2_ oxide is tetragonal structure; at 2370 °C ~ 2706 °C, ZrO_2_ oxide has a cubic structure, and the proportion of three different structures in the weld metal is 5.68:6.10:6.27. Among them, the tetragonal structure of ZrO_2_ oxide is metastable, and it is easy to change into monoclinic structure under the action of external force. In the process of transformation, with 8% volume expansion, the slag is easy to separate from the weld metal surface and has good slag removal performance. However, when the Zr content of weld metal increases, the cubic structure of ZrO_2_ oxide will increase, and the FeO on the weld metal surface is also cubic structure, the binding force between the two is large, resulting in poor slag removal performance, reducing the weld slag removal rate.

### 3.2. Effect of Zr Content on Microstructure of Weld Metal

**Figure 6:**
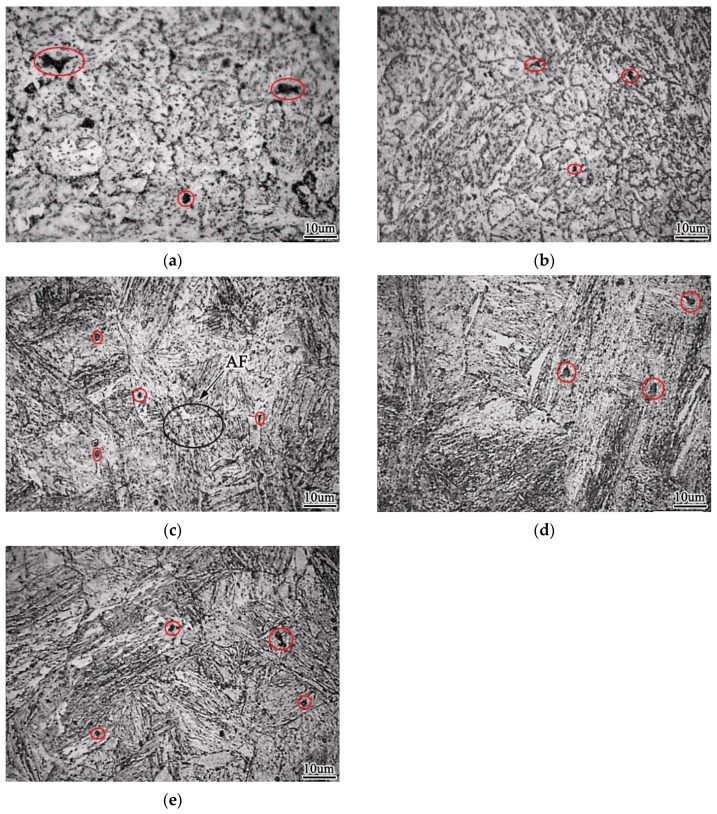
Effect of Zr contents on morphology of weld metal (**a**) 0% Zr (**b**) 0.0028% Zr (**c**) 0.0061% Zr (**d**) 0.0087% Zr (**e**) 0.0135% Zr.

Figure 6 shows the influence of Zr element content on the microstructure of weld metal. It can be seen that when the weld metal does not contain Zr element, the structure is mainly proeutectoid ferrite and granular bainite, with a small amount of acicular ferrite and pearlite, as shown in Figure 6a. There are a lot of inclusions in the weld metal matrix, the particle size of inclusions is large and uneven, and the grain size of weld metal matrix is also relatively large. When the weld metal contains 0.0028% Zr element, the content of eutectoid ferrite and granular bainite in the weld metal structure decreases, and equiaxed fine-grained ferrite and acicular ferrite begin to precipitate. The particle size of inclusions is large but the distribution is uniform, as shown in Figure 6b. When the weld metal contains 0.0061% Zr, the content of acicular ferrite in the weld metal increases, the content of equiaxed fine-grained ferrite begins to decrease, and there is a small amount of pearlite, the particle size of the weld metal is small, and the inclusion particle size is small, showing dispersion distribution, as shown in Figure 6c. When the weld metal contains 0.0087% Zr, the acicular ferrite structure begins to decrease, the equiaxed fine-grained ferrite continues to decrease, the number of eutectoid ferrite increases, and the distribution of inclusions is uneven, as shown in Figure 6d. When the weld metal contains 0.0135% Zr, the acicular ferrite is greatly reduced, there are a large number of granular bainite, a small number of pearlite and proeutectoid ferrite in the structure, and the particle size of inclusions is large and the distribution is uneven, as shown in Figure 6e. The results show that with the increase of Zr content in the weld metal, the content of acicular ferrite and equiaxed fine ferrite in the microstructure increases first and then decreases. When 0.0061% Zr is contained, the acicular ferrite content in the weld metal is the highest, and there is a certain amount of equiaxed fine ferrite, and the content of proeutectoid ferrite is the lowest.

Acicular ferrite is a product of medium temperature transformation and is often included in bainite transformation. It is generally believed that acicular ferrite is composed of many fine non- parallel ferrite lath bundles. The lath bundles are staggered and radiated with an average grain size of 4 μm ~ 5 μm, a length width ratio of 4:1 ~ 8:1, and an angle of about 20° between acicular ferrite laths, so it is a large angle grain boundary [20,21].

When there are cracks in the weld metal, the crack growth near the large angle grain boundary is blocked, and the expanding crack will be blocked and stagnated with the continuous increase of the applied load. At this time, a large number of high-density dislocations in acicular ferrite will gather at the grain boundary, resulting in the existence of internal stress at the grain boundary, which will cause the dislocation inside the grain to produce cross slip, but the direction of dislocation movement There is a certain angle with the original crack direction, that is to say, a new crack is produced. With the further increase of stress, the original crack and the new crack connect each other, and change the direction of crack growth slowly. Therefore, the large angle grain boundary has a blocking effect on the crack growth, which makes the crack difficult to expand.

**Figure 7:**
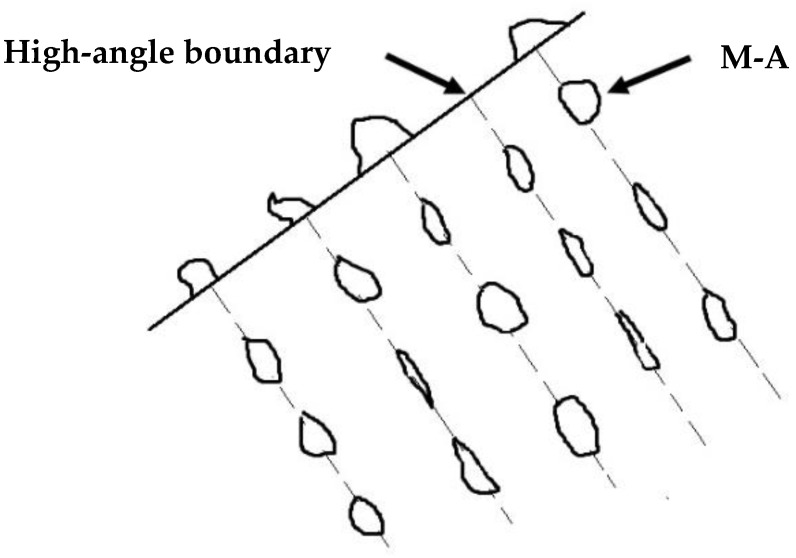
Schematic drawings showing the distribution of M-A in acicular ferrite.

Under the high-resolution TEM, there are many substructures in acicular ferrite, which are M-A componentd and high density dislocationd. The formation of M-A component is due to the formation of particles in austenite that are favorable for acicular ferrite nucleation during the transformation of weld metal, which hinders the growth of austenite ferrite interface. Too much carbon is segregated around the grain boundary. During the subsequent cooling process, austenite has no time to transform into ferrite, but martensite transformation occurs to form martensite. These martensites and carbon rich residual are formed The remaining austenite forms the M-A component, as shown in Figure 7.

**Figure 8:**
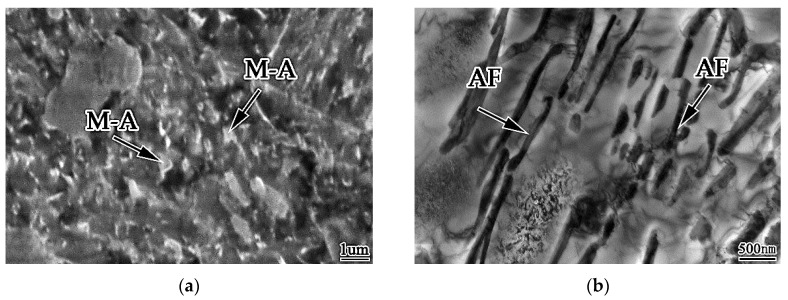
Morphology of M-A in acicular ferrite (**a**) SEM photograph (**b**) TEM photograph.

Under the scanning electron microscope, the M-A component is uniform bright white tissue with slight convexity, as shown in Figure 8a. Under the transmission electron microscope, the details can be clearly seen, as shown in Figure 8b. 

The alloy elements can increase the austenite stability and make the distribution of acicular ferrite lath more obvious, and the M-A component is distributed between acicular ferrite laths in a strip shape. A proper increase of cooling rate can reduce the amount of M-A component in acicular ferrite, and make it fine and dispersive. When the cooling rate increases, the M-A component increases and its size increases, which is rod-shaped and massive; when the cooling rate slows down, the austenite island will decompose into ferrite and cementite, such as upper bainite. The M-A component in acicular ferrite not only improves the strength of weld metal, but also has a good effect on toughness. This is because the M-A component is hard brittle phase, which makes acicular ferrite have higher hardness, which is beneficial to improve the strength of weld metal.

**Figure 9:**
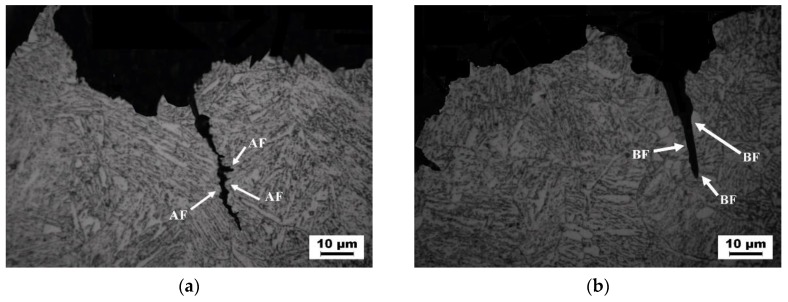
Crack extension of impact fracture of weld metal. (**a**) across the AF (**b**) across the BF.

Figure 9 shows the longitudinal section morphology of weld metal impact fracture. It can be seen that when the crack extends to the acicular ferrite in the weld metal, the crack extension path becomes zigzag, the crack extends along the acicular ferrite boundary, and the path is curved, as shown in Figure 9a; When the crack extends to upper bainite, the crack extension path becomes straight, and the crack penetrates the interior of the upper bainite, the propagation resistance is small, as shown in Figure 9b. When the weld metal cracks, the crack extends near the M-A component, the dislocation produced by the crack will be blocked by the M-A component and bent, and the dislocation will gather around the M-A component to form dislocation clusters. The dislocation cluster is pinned by M-A component, which hinders the crack growth and changes its direction. When the applied stress increases, the cracks will join together and cross the M-A component, and resume the original direction of propagation and continue to expand forward. Therefore, the existence of M-A component in acicular ferrite will make the dislocation produced by the crack fixed, affect the movement of the crack and change the direction of the crack, so as to improve the toughness of acicular ferrite.

### 3.3. Effect of Zr on the Hardness of Weld Metal

**Figure 10:**
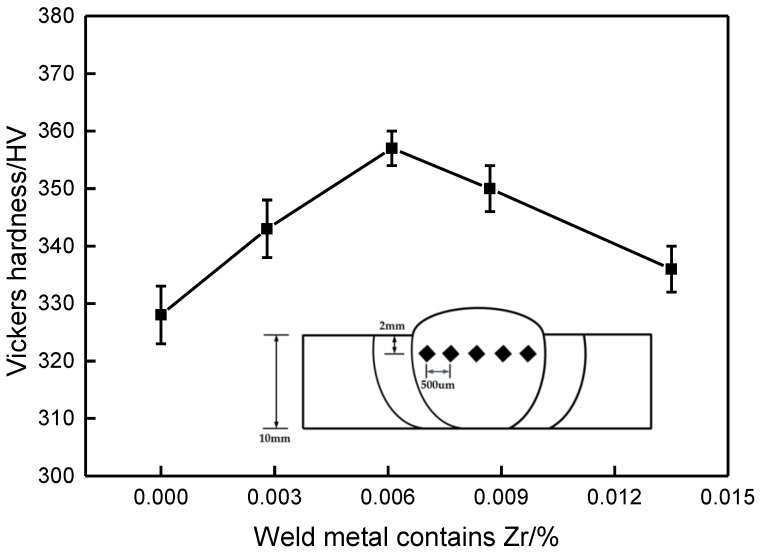
Effect of Zr content on vickers hardness of weld metal.

Figure 10 shows the effect of Zr content on the hardness of weld metal. It can be observed that when Zr content is 0, the weld hardness is the lowest. 

With the increase of Zr content, the hardness increased gradually. When Zr content was 0.0061%, the hardness reached the maximum value of 357HV. With the increase of Zr content, the hardness began to decline. Basing on the analysis of Figure 6, when Zr content is 0, coarse microstructure, acicular ferrite is less and distributed unevenly, and the hardness is low. With the increase of Zr content, the shape and distribution of acicular ferrite grains become more reasonable while refining, and the hardness value increases obviously. When the Zr content further increase, there are a large number of granular bainite, a small number of pearlite and proeutectoid ferrite in the structure, so the hardness was reduced.

### 3.4. Effect of Zr on Tensile Properties of Weld Metal

**Figure 11:**
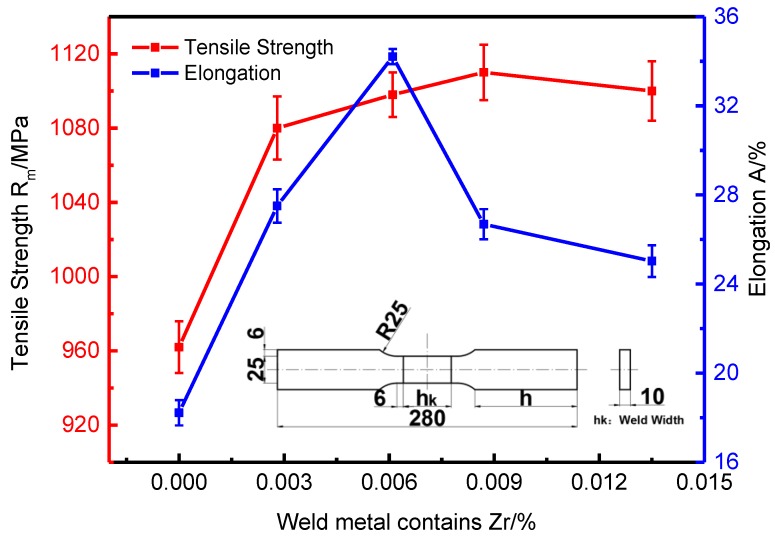
Effect of zirconium content on tensile strength and elongation of welded joints.

Figure 11 shows the effect of Zr content of weld metal on tensile properties. It can be seen that when the Zr content of weld metal is 0, the tensile strength and elongation of weld metal were low; with the increase of Zr content of weld metal, the tensile strength and elongation of weld metal show a trend of increasing first and then decreasing. When the weld metal contains 0.0061% Zr, the elongation of the weld metal reaches the maximum value of 34%, the percentage elongation after fracture begins to decline with the increase of Zr content, the plastic toughness of the weld metal becomes worse, but the strength of the weld metal continues to increase. When the Zr content of weld metal reaches 0.0087%, the tensile strength of weld metal reaches the maximum value of 1110 Mpa, and then continues to increase the Zr content, and the tensile strength of weld metal begins to decline.

**Figure 12:**
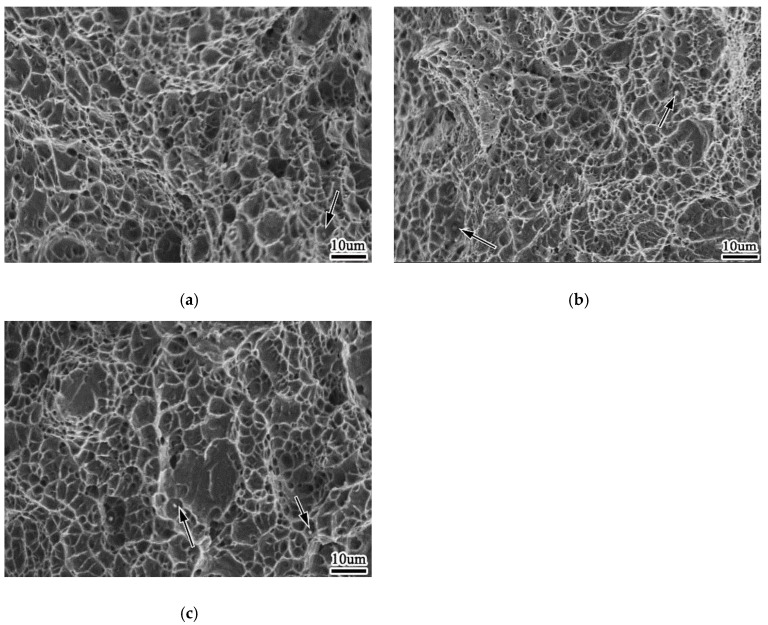
Morphology of tensile fracture of weld metal (**a**) 0%Zr (**b**) 0.0061%Zr (**c**) 0.0135%Zr.

Figure 12 is SEM picture of the tensile fracture of 0% Zr, 0.0061% Zr and 0.0135% Zr welds, and the composition analysis of the dimple inclusions indicated by the arrows in the figure is shown in Table 8. It can be seen from the tensile fracture of the weld metal that when the weld metal does not contain Zr element, the tensile fracture is characterized by the cross distribution of large dimple and small dimple, with shallow dimple depth, and the inclusions are very few; when the weld metal contains 0.0061% Zr, the radial zone of the fracture is characterized by small dimple, with deep dimple depth; when the weld metal contains 0.0135% Zr, the tensile fracture is characterized by large dimple, which is divided around the large dimple Small dimples are distributed, and the depth of each dimple is shallow. The depth of fracture dimple is closely related to the plastic deformation ability of weld metal. At the same time, the quantity of inclusions increases and the distribution is uniform. When the plasticity of weld metal is large, the dimple depth is deep; otherwise, the dimple is shallow. The size of fracture dimple depends on the size and quantity of the second phase particles. When the particle size of the second phase is large, the dimple size is large; when the particle number of the second phase is small, the dimple size is large. The size of inclusion is generally 0.5–1 um.

According to Figure 6 and Figure 10, when the weld metal does not contain Zr, the weld metal structure is mainly composed of proeutectoid ferrite and granular bainite, with coarse structure and poor weld metal strength and plastic toughness. With the increase of Zr content in the weld metal, the amount of proeutectoid ferrite and granular bainite in the weld metal decreases, the amount of acicular ferrite increases, the microstructure is fine, and there are high density dislocations, so the weld metal has high strength and better plastic toughness. When the Zr content in the weld metal continues to increase, the arc will be unstable during welding, and the contact between the droplet and the outside air will be increased. The weldability will be poor, and the structure is mainly bainite. Therefore, the strength and plastic toughness of the weld metal are poor.

The compositions of inclusions in 0% Zr, 0.0061% Zr and 0.0135% Zr were measured, each group measured five inclusions, each group lists one inclusion composition in Table 8. It can be seen from Table 8 that with the change of Zr content in weld metal, the composition of micro inclusions in fracture is also different, and the change of composition and quantity of inclusions has an impact on the size of inclusions, thus affecting the performance of weld metal. When the weld metal contains 0% Zr, the main components of inclusions in tensile fracture are Fe, Al, Mg, O and N, while Al and Ti are strong deoxidizing and nitrogen fixing elements, indicating that the inclusions are metal oxides and nitrides. The results show that AlN exists in the inclusions, and AlN is a hexagonal inclusion, which have a cutting action on the weld metal. With the increase of Zr content in weld metal, the content of Zr in inclusions also increases, and the main component of inclusions changes to Fe. Compared with inclusions without Zr, the content of n decreases significantly. When the Zr content of the weld metal reaches 0.0135%, the main components of inclusions are Fe and Zr, and the contents of Al and N increase again. It is found that the Zr content in inclusions is much higher than that in the weld metal matrix, which indicates that Zr is more likely to exist in inclusions than in the solid solution formed by the weld metal.

### 3.5. Effect of Zr Elements on Impact Toughness of Weld Metal

**Figure 13:**
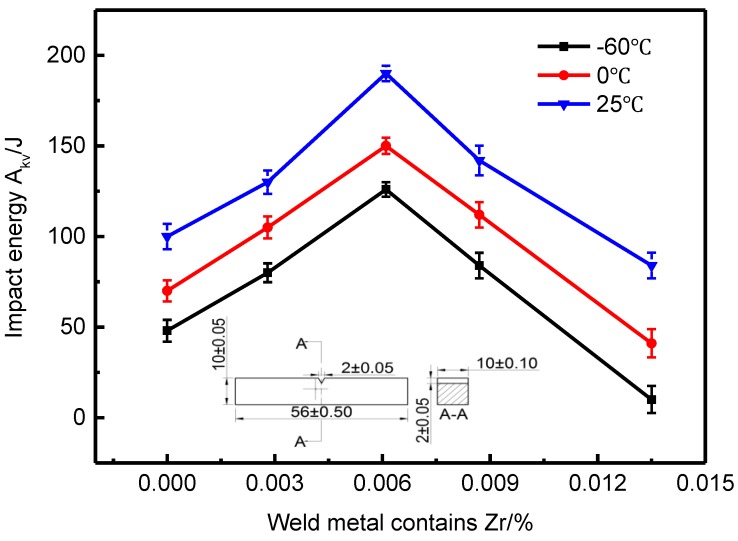
Effect of Zr contents on impact energy of weld metal.

Figure 13 shows the effect of Zr content of weld metal on impact energy of weld metal. It can be seen that with the increase of Zr content, the impact energy of weld metal increases first and then decreases at room temperature and low temperature, and the maximum value appears at 0.0061% Zr. When Zr content is 0%, the average low-temperature impact energy of weld metal at –60 °C is 48 J; when Zr content is 0.0061%, the average low-temperature impact energy of weld metal at –60 °C is 126 J; when Zr content is 0.0135%, the average low-temperature impact energy of weld metal at –60 °C is 10 J, even lower than the impact energy of weld metal without Zr element. This is because when the Zr content is very high, the acicular ferrite structure can be formed, but the C content of the untransformed austenite increases due to the discharge of C from the ferrite to the austenite. During the cooling process, part of the austenite transforms into martensite, becoming the brittle phase of M-A component, which is easy to become the source of cracks, leading to the decrease of the impact toughness of the weld metal.

**Figure 14:**
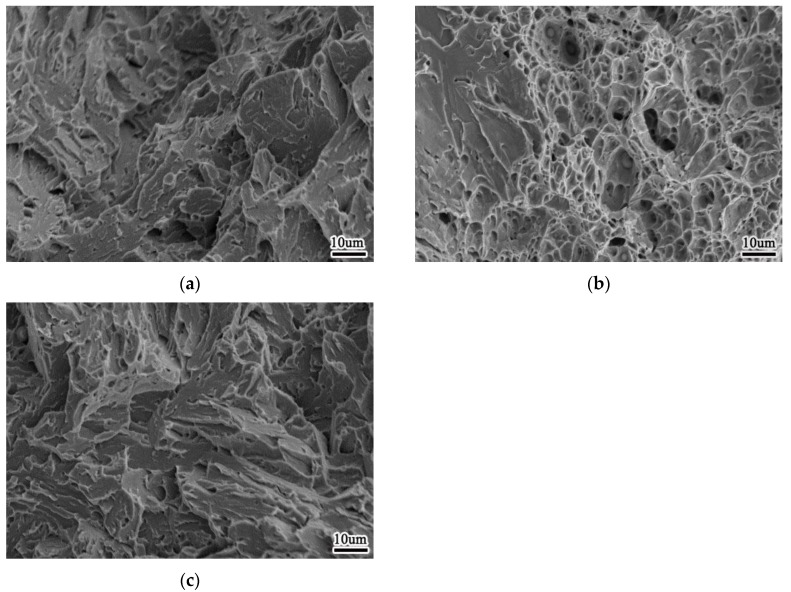
Morphology of impact fracture of weld metal at −60 °C (**a**) 0%Zr (**b**) 0.0061%Zr (**c**) 0.0135%Zr.

Figure 14 shows the impact fracture morphology of –60 °C weld metal when Zr content of weld metal is 0%, 0.0061% and 0.0135%, respectively. When the Zr content of the weld metal is 0%, the radial area of the impact fracture of the weld metal is cleavage fracture, showing a fan-shaped pattern; when the Zr content of the weld metal is 0.0061%, the impact fracture of the weld metal contains a large number of dimples, showing a ductile fracture; when the Zr content of the weld metal is 0.0135%, the impact fracture shows a brittle fracture feature, with poor impact.

According to Figure 6 and Figure 13, when the Zr content of weld metal is 0%, the microstructure of weld metal is proeutectoid ferrite and granular bainite, the microstructure is relatively coarse, which is unfavorable to the impact toughness of weld metal. With the increase of Zr content in the weld metal, the amount of proeutectoid ferrite and granular bainite in the structure decreases, the amount of acicular ferrite increases, and the structure becomes smaller. Because acicular ferrite has high density dislocation and substructure, it has strong crack growth resistance, which makes the weld metal have good impact toughness. When the Zr content of the weld metal reaches a high level, the weld metal is composed of granular bainite with coarse grains, which has a negative impact on the impact toughness of the weld metal.

### 3.6. Strengthening and Toughening Mechanism of Weld Metal of High Strength Steel

In order to improve the strength and toughness of the weld metal of high strength steel, on the one hand, it is necessary to add alloy elements into the weld metal, change the composition of the weld metal, obtain ideal structure, and increase enough resistance to dislocation movement, on the other hand, it is necessary to consider that when dislocation movement occurs, the matrix fabric should maintain enough plasticity.

**Figure 15:**
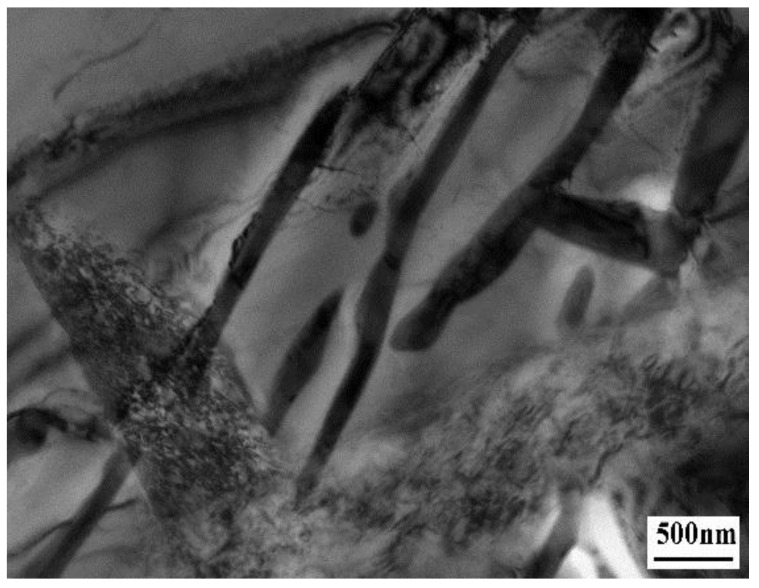
TEM morphology of acicular ferrite in 0.0061% Zr weld metal.

The transmission experiment was carried out on the best specimen (0.0061% Zr). Figure 15 shows the microstructure of acicular ferrite by TEM in this test. At this time, the microstructure of weld metal is 1.2% Mn, 0.31% Mo, 0.35% Si, 0.67% Ni, 0.53% Cr, 0.21% Al, 0.35% Ti, 0.005% B and 0.0061% Zr. It can be seen from the above that acicular ferrite is a series of interleaved nonparallel needle like structures woven together, with large angle grain boundary and high density dislocation, and the angle between adjacent grain boundaries is greater than 15°. Therefore, when microcracks in weld metal propagate between interlaced acicular ferrite, the path is zigzag, which requires more energy consumption, leading to the improvement of strength and toughness.

According to the theory derived by Bhadeshia, the relationship between the number of dislocations and the transition temperature in shear transformation is as follows [22]:

(3) $$\ln\rho =21.3772+15843.4663/T-4099430/T^{2}$$

where *T* is the transition temperature, unit: K; *ρ* is the dislocation density, and the unit is m^2^.

As the transition temperature of sideboard ferrite is between 1023 K and 923 K, the dislocation density is 2 × 10^14^ ~ 4 × 10^14^ from Equation (3) [23]; the transition temperature of acicular ferrite is between 873K and 723K, and the dislocation density is between 6 × 10^14^ ~ 2 × 10^15^. The density of acicular ferrite is much higher than that of sideboard ferrite, so the strength of acicular ferrite is much higher than that of sideboard ferrite.

In the tensile test, no obvious yield point was found in the tensile process. This is because although acicular ferrite has high-density dislocation, because the C content of the welding material itself is very low, there is not enough C to lock the dislocation movement, so has is a body centered cubic structure, with high stacking fault energy, it is not easy to generate extended dislocations and lead to cross slip, so the dislocations have great mobility.

**Figure 16:**
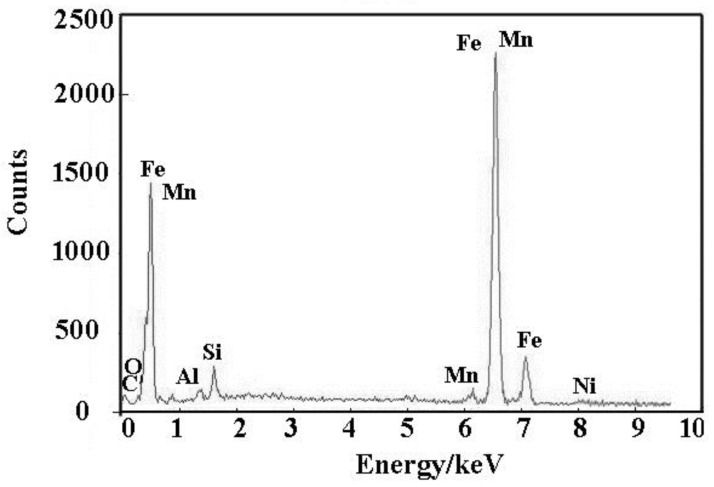
Energy spectrum analysis of acicular ferrite in 0.0061% Zr weld metal.

Figure 16 is an energy spectrum analysis of the acicular ferrite lamellar matrix of Figure 15. It can be seen that the content of Mn, Ni and other elements in acicular ferrite matrix is high, which indicates that these elements can indeed enter the matrix to play a role of solid solution strengthening.

According to the cottrel energy analysis method, the formula of critical stress required for crack growth is as follows [24]:

(4) $$\sigma_{c}=2G\gamma_{S}K_{y}/\sqrt{d}$$

where *σ*_c_ is the nominal fracture stress of the crack, MPa; *G* is the modulus of elasticity, MPa; *γ*_s_ is the surface energy per unit area of the crack, MJ/m^2^; *k*_y_ is the dislocation pinning coefficient; *d* is the grain diameter, mm. It can be seen that with the decrease of grain size, the critical stress required for crack growth becomes larger, and the plastic toughness of the material is better.

It can be seen from Equation (4) that to improve the plastic toughness of the material, the value of *G*, *k*_y_ *γ*_s_ can be increased, or the value of *d* can be reduced. Because both the *G* and *k*_y_ values are related to the physical quantities of the material, when the material is determined, the critical stress required for crack growth can be increased only by reducing the *d* value, i.e., refining the grains.

The ductile brittle transition temperature between *T*_c_ and ln*d*^−1/2^ is shown in Equation (5) [25]:

(5) $$T_{c}=K-\beta \ln d^{-1/2}$$

where, *K* and *β* are constants and *d* is grain size. 

By adding microelement Zr, Zr could spheroidize and refine the inclusions in the weld metal, it could also increase the inclusions which were suitable for inducing acicular ferrite nucleation and increase the number of acicular ferrite. With the increase of Zr content in the weld metal, the weld metal microstructure was gradually refined, the pre eutectoid ferrite at the grain boundary was inhibited, and a large number of fine and uniform acicular ferrite appear in the grains. It can be seen from Formula (5) that there is a linear relationship between the ductile brittle transition temperature *T*_c_ and the grain size ln*d*^−1/2^. That is to say, refining acicular ferrite layer can reduce the ductile brittle transition temperature and improve the low-temperature impact toughness of weld metal.

## 4. Conclusions

In this paper, taking Fe Mn Mo Cr Ni as the main alloy system and BaF_2_-CaF_2_-Al-Mg as the basic slag system, the influence rule and mechanism of Zr element in welding wire powder on the weld metal structure and mechanical properties are studied. The following conclusions are obtained:

- With the increase of Zr content, the welding spatter rate first decreased and then increased, and the change trend of slag removal rate was the opposite. When the weld metal contains 0.0061% Zr, the minimum spatter rate of weld metal is only 6.92%, and the maximum slag removal rate is 96.7%. The ZrO_2_ produced during welding will increase the surface tension of the droplet, and the spatter rate is small. The tetragonal metastable structure of ZrO_2_ is easy to become monoclinic structure under the external forces, the process of structural transformation will make the volume expand by 8% and the deslagging property is good.
- When the weld metal contains 0.0061% Zr, the microstructure of the weld metal contains a lot of acicular ferrite, few pores, small inclusions and dispersion distribution. However, when the Zr content of the weld metal is too high, the stability of the welding arc becomes poor. When welding, the external air is easy to enter the arc atmosphere and contact with the droplet, which reduces the protection effect of the arc, resulting in the poor structure of the weld.
- With the increase of Zr content in weld metal, the hardness, tensile strength, elongation and impact energy of weld metal all increased first and then decreased. When the weld metal contains 0.0061% Zr, the comprehensive properties of the weld metal is the best, the hardness value is 357 HV, the tensile strength is 1098 Mpa, the elongation is 34%, and the low-temperature impact energy at −60 °C is 126 J. The existence of M-A in acicular ferrite will fix the dislocation produced by the cracks, affect the movement of the cracks and change the direction of the cracks, so as to improve the toughness of acicular ferrite.
- The ideal weld metal structure of 960 Mpa high-strength steel is acicular ferrite with fine grains. At the same time, alloy elements such as Mn, Mo, Ni, Cr and Si can be used for solution strengthening and precipitation strengthening, Ti and B elements can be used for pinning to refine grains and grain boundary strengthening, and Al and Zr elements can be used to increase acicular ferrite nucleation point, so as to jointly improve the strength and improvement of weld metal Low temperature impact toughness.

## Figures and Tables

**Table 1:** Chemical composition of Q960 high strength steel (mass, wt %).

Component	C	Si	Mn	Cr	Ni	Mo	Nb	Ti	B	S	P
Content	0.18	0.5	1.6	0.8	1.0	0.6	0.05	0.03	0.005	0.002	0.011

**Table 2:** Mechanical properties of Q960 high strength steel.

Property	Tensile Strength Rm/MPa	Elongation A/%	Impact Energy Akv (−20 °C)/J
Value	≥960	>12	≥30

**Table 3:** Chemical composition of steel strip (mass, wt %).

Component	C	Si	Mn	P	S	Fe
Content	0.03	0.01	0.18	0.013	0.011	Bal.

**Table 4:** Powder formula of flux cored wire (mass, wt %).

Component	Zr	Mn	Si	Ni	Mo	Cr	Ti	B	Al	Fe
Content	0.000	1.20	0.20	0.60	0.40	0.60	0.25	0.005	0.20	Bal.
	0.004	1.20	0.20	0.60	0.40	0.60	0.25	0.005	0.20	Bal.
	0.008	1.20	0.20	0.60	0.40	0.60	0.25	0.005	0.20	Bal.
	0.012	1.20	0.20	0.60	0.40	0.60	0.25	0.005	0.20	Bal.
	0.016	1.20	0.20	0.60	0.40	0.60	0.25	0.005	0.20	Bal.

**Table 5:** Mass fraction of test powder (mass, wt %).

Component	Electrolytic Manganese	Nickel Powder	Molybdenum Powder	Metal Chromium	Zirconium Powder	Aluminum Powder	Iron Powder
Purity	99	99.5	99.9	99.5	99	99.99	99.5

**Table 6:** Welding process parameters.

Layer	Wire Feed Rate m/min	Welding Voltage U/V	Welding Current I/A	WELDING Rate mm/min	Wire Extension L/mm	Interpass Temperature /°C
1 pass	2	28~30	200~250	230	18~20	150 ± 10
2–5 pass	2	30~32	250~300	230	18~20	150 ± 10
6–7 pass	2	28~30	200~250	230	18~20	150 ± 10

**Table 7:** Chemical composition of weld metal (mass, wt %).

Component	Zr	Mn	Si	Mo	Cr	Ni	Ti	B	Al	O	N
Content	0.0000	1.09	0.37	0.85	0.89	1.19	0.29	0.002	0.18	0.016	0.028
	0.0028	1.14	0.35	0.80	0.90	1.18	0.33	0.006	0.16	0.016	0.019
	0.0061	1.23	0.33	0.82	0.72	1.16	0.28	0.004	0.18	0.015	0.020
	0.0087	1.21	0.38	0.88	0.90	1.20	0.30	0.003	0.19	0.010	0.017
	0.0135	1.20	0.36	0.82	0.88	1.20	0.29	0.003	0.19	0.007	0.014

**Table 8:** Inclusion composition of tensile fracture.

Component	Mn	Ti	Mg	Al	O	N	Zr	Fe
0% Zr	0.8	1.8	12.4	18	13.2	9.5	0	40.7
0.0061% Zr	1.1	0.6	1.8	4.7	4.7	0.1	1.2	80.5
0.0135% Zr	0	1.1	0.65	7.1	3.2	5.2	3.3	79.7
